# Advances in the axenic isolation methods of *Blastocystis* sp. and their applications

**DOI:** 10.1017/S0031182023001300

**Published:** 2024-02

**Authors:** Xuefang Mei, Lai Wei, Changwei Su, Zhenke Yang, Xiaowei Tian, Zhenchao Zhang, Shuai Wang

**Affiliations:** Xinxiang Key Laboratory of Pathogenic Biology, Department of Pathogenic Biology, School of Basic Medical Sciences, Xinxiang Medical University, Xinxiang, Henan, PR China

**Keywords:** antibiotics, axenic isolation, *Blastocystis* sp, influencing factors, medium

## Abstract

*Blastocystis* sp. is a prevalent protistan parasite found globally in the gastrointestinal tract of humans and various animals. This review aims to elucidate the advancements in research on axenic isolation techniques for *Blastocystis* sp. and their diverse applications. Axenic isolation, involving the culture and isolation of *Blastocystis* sp. free from any other organisms, necessitates the application of specific media and a series of axenic treatment methods. These methods encompass antibiotic treatment, monoclonal culture, differential centrifugation, density gradient separation, micromanipulation and the combined use of culture media. Critical factors influencing axenic isolation effectiveness include medium composition, culture temperature, medium characteristics, antibiotic type and dosage and the subtype (ST) of *Blastocystis* sp. Applications of axenic isolation encompass exploring pathogenicity, karyotype and ST analysis, immunoassay, characterization of surface chemical structure and lipid composition and understanding drug treatment effects. This review serves as a valuable reference for clinicians and scientists in selecting appropriate axenic isolation methods.

## Graphical abstract

**Figure:**
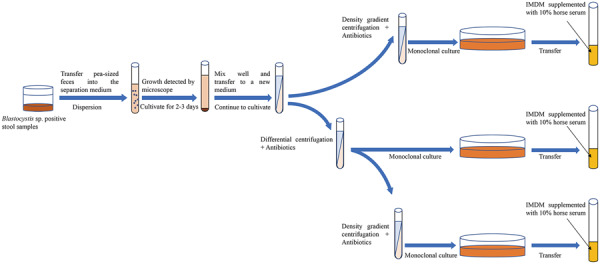

## Introduction

*Blastocystis* sp. is a common intestinal protist found in humans and animals (Udonsom *et al*., 2018), and is distributed worldwide, encompassing both urban and rural settings across developed and developing countries (Scanlan *et al*., 2014; Udonsom *et al*., 2018). In Poland, infection rates range from 0.14 to 23.6% across various study groups from 1955 to 2022 (Rudzińska and Sikorska, 2023). This figure, meanwhile, often exceeds 50% in developing countries and even 100% among children in Senegal River Basin (Alfellani *et al*., 2013; El Safadi *et al*., 2014; Popruk *et al*., 2021). *Blastocystis* sp. is transmitted through the fecal–oral route, and people or animals are infected by ingesting contaminated food or water (Maloney *et al*., 2019). Although some cases are asymptomatic, *Blastocystis* sp. infection is closely linked to conditions such as irritable bowel syndrome, terminal ileitis and ulcerative colitis, manifesting mainly as abdominal pain, diarrhoea, flatulence and constipation (Tunalı *et al*., 2018; Basuony *et al*., 2022).

The morphological diversity of *Blastocystis* sp. includes vacuolar, granular, amoebic, cyst, avacuolar and multivacuolar forms (Stenzel and Boreham, 1996). Among these, the vacuolar form is the most prevalent and typical form of *Blastocystis* sp., which is often used as the classical morphological standard of *Blastocystis* sp. infection. However, the presence of abundant associated bacteria poses challenges for ultrastructural observation of *Blastocystis* sp. through an electron microscope (Zierdt and Williams, 1974).

Isolating *Blastocystis* sp. from fecal cultures often introduces bacterial contamination, rendering many animal experimental results unacceptable or leading to inconsistencies across studies. Axenic strains, free from contaminating organisms, facilitate more scientific and persuasive in-depth studies on various aspects of *Blastocystis* sp., such as karyotype modelling, antigen analysis, enzyme activity and surface biochemical components, thereby advancing our understanding of the biological aspects of *Blastocystis* sp. (Lanuza *et al*., 1996*b*). Since the successful axenic culture of *Blastocystis* sp. by Zierdt and Williams in 1974, numerous scholars have attempted axenic isolation methods.

This paper builds upon previous studies, reflecting a deep understanding of *Blastocystis* sp. and the significance of its axenic culture. The review encompasses presently available axenic isolation techniques and factors influencing their applicability, emphasizing the importance of axenic isolation for studying *Blastocystis* pathogenicity, describing its surface biochemical components and elucidating therapeutic effects. The content serves as a valuable reference for clinicians and scientists in selecting appropriate axenic isolation methods.

## The axenic cultivation of *Blastocystis* sp.

Axenic cultivation of *Blastocystis* sp. involves the culture of *Blastocystis* sp. free from contamination with other organisms. This necessitates the application of a suitable culture medium and a series of axenic treatment methods, with the selection of the optimal culture medium being crucial (Tan *et al*., 2000). An efficient and economical medium for isolating *Blastocystis* sp. serves as the foundation for subsequent studies on factors such as its life history, morphological classification and infection immunity (Tan, 2008). Axenic methods include antibiotic treatment, monoclonal culture, differential centrifugation, density gradient separation, micromanipulation and the combined application of culture medium. Obtaining axenic *Blastocystis* sp. often requires the combined application of different methods, presenting a promising avenue for further research.

### Commonly used media for the axenic isolation of *Blastocystis* sp.

The *in vitro* culture of *Blastocystis* sp. involves the utilization of monophasic and biphasic media. The biphasic medium comprises 2 media with distinct physical states, while the monophasic medium consists of only 1 physical medium. Based on physical state, the medium is categorized as solid, liquid and solid–liquid biphasic. The solid medium contains agar, and the liquid medium includes Jones medium, Iscove's modified Dulbecco's medium (IMDM) and Dulbecco's modified Eagle medium (DMEM). Typically, a single-phase medium is prepared by sequentially adding necessary raw materials, sterilizing the mixture under high pressure and then packaging it in suitable volumes according to experimental requirements. Biphasic medium includes Locke's egg serum (LES) medium and Boeck–Drbohlav biphasic modified medium (BDMM), composed of solid and liquid components. The preparation involves configuring the solid phase first, followed by the liquid phase and finally covering the solid medium's surface with the liquid medium. Table 1 provides details of specific culture media.

**Table 1.** Commonly used media for aseptic isolation

Physical state	Culture medium	Culture medium configuration	Reference
Monophasic medium	IMDM	Commercial medium.	Ho *et al*. (1993); Ng and Tan (1999)
	Agar	Equal amounts of 2× IMDM and 0.72% agar mixed in a 50 mL centrifuge tube, and different final volumes obtained according to the experimental requirements; an appropriate amount of serum and *Blastocystis* sp. inoculation then added and cultured in an anaerobic tank at 37°C.	Valido and Rivera (2007)
	Modified Jones medium	93.8 mL Na_2_HPO_4_–9.46 g L^−1^ of distilled water, 31.3 mL KH_2_HPO_4_–9.08 g L^−1^ of distilled water, 562.5 mL NaCl–9 g L^−1^ of distilled water, 0.1% yeast extract are mixed and packed in 12 mL Falcon tubes; an appropriate amount of serum and *Blastocystis* sp. added and cultured under anaerobic conditions at 37°C.	Lepczyńska and Dzika (2019)
	DMEM	Commercial medium.	Karamati *et al*. (2020)
Biphasic medium	LES medium	Equal volume of Locke's solution added to 30° egg slope medium, sterilized under high pressure; 20% sterile serum (human, horse, chicken, monkey and calf), 2 mL mineral oil covering layer, an appropriate amount of antibiotics added; medium placed in a CO_2_ incubator and cultured at 37°C.	Zierdt and Williams (1974)
	BDMM	Locke solution configuration: each litre of distilled water contains 5 g glucose, 16 g sodium chloride, 0.4 g calcium chloride, 0.4 g potassium chloride, 0.02 g magnesium chloride, 4 g sodium phosphate dibasic, 0.8 g sodium bicarbonate and 0.6 g potassium dihydrogen phosphate. The solution is boiled, allowed to sit overnight, filtered through number 1 Whatman paper, mixed with 0.02% phenol red (1:25, v/v), sterilized under high pressure and kept at 4°C until use. All eggs homogenized in Locke solution for the solid medium. The yolk suspension distributed in a 16 × 160 mm^2^ crystal spiral cover tube with the amount of 5 mL, concentrated at an inclination of 30° at 80°C for 30 min, sterilized under high pressure and stored at 4°C. The liquid phase medium consists of a mixture of Locke solution and inactivated (56°C, 30 min) fetal horse or calf serum (1:1, v/v). The liquid phase is temporarily prepared. The complete culture medium prepared by adding 4 mL liquid medium to each tube, and the medium pre-reduced under anaerobic condition for at least 48 h before use.	Lanuza *et al*. (1996*b*)
	Modified BDMM	Modified BDMM was improved from BDMM, which is composed of egg slant and Locke solution covered with the appropriate amount of serum.	Teow *et al*. (1991)

### Methods for the axenic isolation of *Blastocystis* sp.

#### Antibiotic treatment

**Figure 1.**
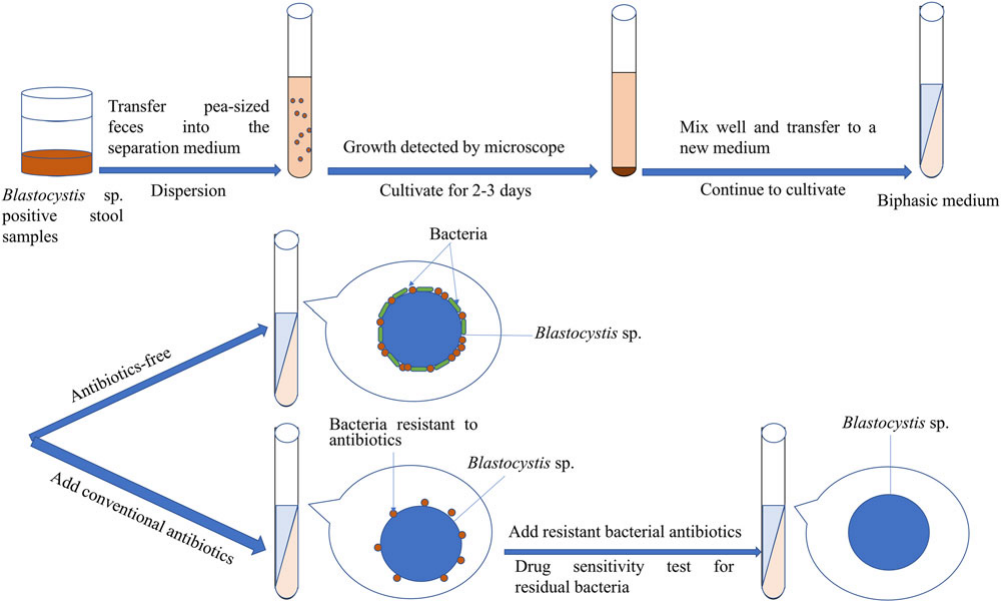
Antibiotic treatment for the axenic isolation of *Blastocystis* sp.

Antibiotic treatment has been a key approach for the axenic isolation of *Blastocystis* sp. since the seminal study by Zierdt and Williams (1974). The inclusion of antibiotics in the culture medium (Fig. 1) has become a widely employed method, although it presents challenges. While antibiotics contribute to asepsis, complete elimination of bacteria may not be achieved due to the potential development of antibiotic resistance among the microbial population. It is important to note that low bacterial counts might be mistakenly regarded as axenic (Ng and Tan, 1999). Ampicillin and streptomycin combination stands out as a straight forward and effective addition, proving capable of eliminating a significant portion of bacteria (Teow *et al*., 1991, 1992). Additionally, amphotericin is employed to target yeasts and moulds, common contaminants in the initial culture stages (Zierdt and Williams, 1974). Ceftizoxime and vancomycin can eliminate drug-resistant bacteria (Zierdt, 1991). Despite the benefits of antibiotic treatment in achieving asepsis, it comes with the potential drawback of inhibiting parasite growth, with alterations to the size and vacuole content of *Blastocystis* sp. (Teow *et al*., 1992). It is noteworthy that the introduction of antibiotics can impact the longevity of *Blastocystis* sp., with signs of decline observed after the third passage in a chloramphenicol-containing medium (Zierdt *et al*., 1983). The strategic addition of antibiotics poses a delicate balance, as the direct use of a mixture of multiple antibiotics may result in insufficient growth of *Blastocystis* sp., while the sequential addition of antibiotics, 1 at a time, has proven beneficial for promoting growth (Lanuza *et al*., 1996*b*).

#### Monoclonal culture

**Figure 2.**
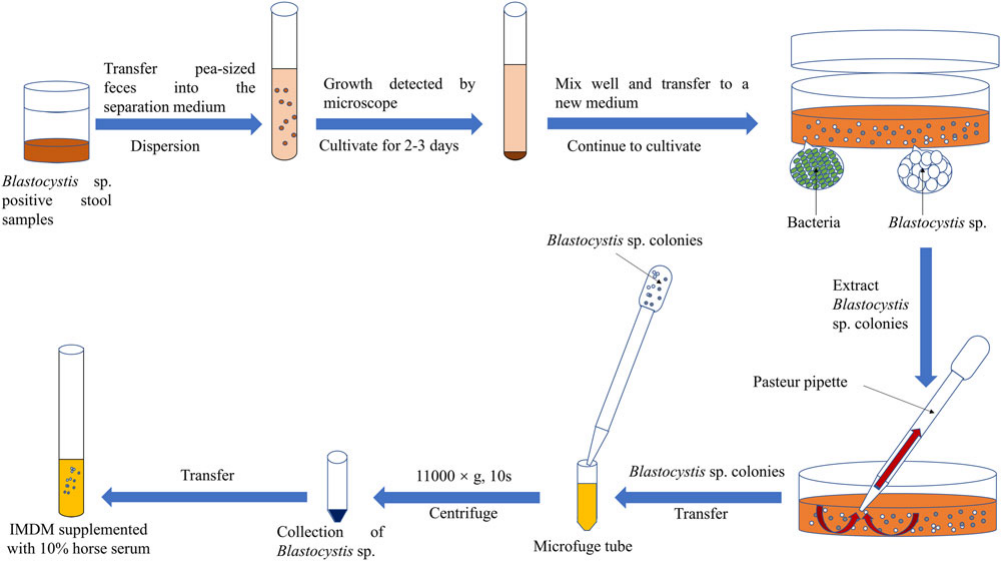
Monoclonal culture for the axenic isolation of *Blastocystis* sp.

Agar cloning technology plays a pivotal role in achieving the physical separation of *Blastocystis* sp. from bacteria, ensuring the asepsis of *Blastocystis* sp. (Valido and Rivera, 2007) (Fig. 2). Monoclonal culture, in the presence of certain bacteria, facilitates the distinct separation of pure *Blastocystis* sp. colonies from bacterial colonies. The pioneering work of Tan *et al*. marked the cultivation of axenic *Blastocystis* sp. colonies in Petri dishes containing soft agar, leading to the successful cultivation of a substantial number of amoebic *Blastocystis* sp. (Tan *et al*., 1996*a*). In the same year, Tan *et al*. introduced sodium thioglycolate to enhance the colony formation efficiency (%CFE = the number of growing colonies/inoculated cells × 100), resulting in the abundant production of *Blastocystis* sp. from a single clone (Tan *et al*., 1996*b*). The monoclonal culture was further applied by Ng and Tan to strains treated with antibiotics, leading to the successful isolation of pure *Blastocystis* isolates (Ng and Tan, 1999).

The advantage of monoclonal culture lies in the physical separation of *Blastocystis* sp. colonies and bacterial colonies, with bacterial colonies growing in 0.36% Bacto agar easily distinguishable from the diffuse *Blastocystis* sp. colonies visible under an inverted microscope. However, this method was associated with the following drawbacks: (1) counting colonies becomes challenging due to the semisolid nature of agar; (2) cloning and growth of *Blastocystis* sp. in agar necessitate various nutrients, requiring the preparation of fresh culture medium and (3) extracting *Blastocystis* sp. colonies proves difficult as they are generally embedded in agar. To address these challenges, Tan *et al*. introduced a simplified and efficient method for clonal growth on solid agar, increasing the agar content from 0.36 to 2%. This modification allowed the successful culture of *Blastocystis* sp. colonies on the agar surface, facilitating their isolation for further studies (Tan *et al*., 2000). It is crucial to note that abundant bacterial colonies can impede the identification and isolation of *Blastocystis* sp. colonies. Therefore, antibiotic treatment is deemed necessary in monoclonal culture to reduce the bacterial population and aid in the isolation of *Blastocystis* sp. colonies (Ng and Tan, 1999; Valido and Rivera, 2007).

#### Differential centrifugation technique

**Figure 3.**
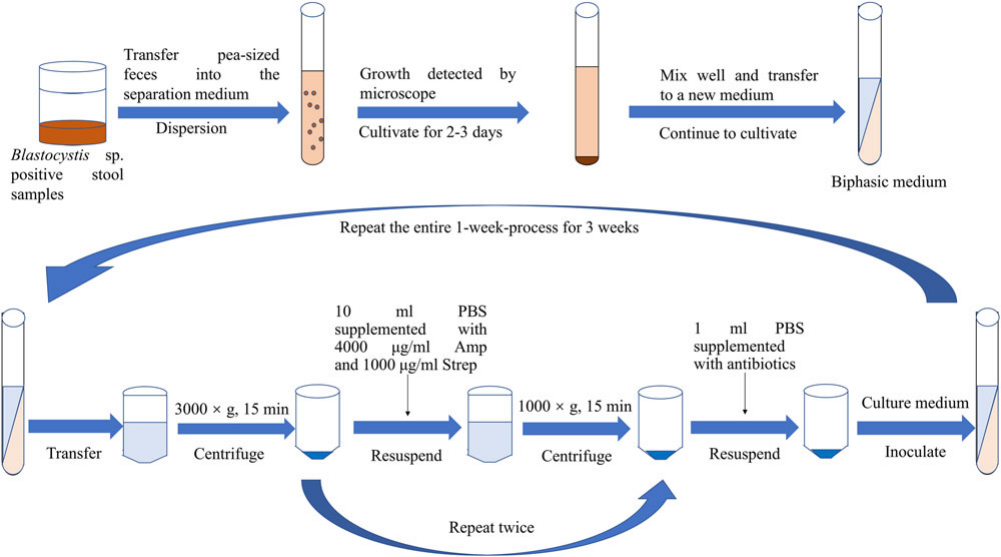
Differential centrifugal technique for the axenic isolation of *Blastocystis* sp. PBS, phosphate-buffered saline; Amp, ampicillin; Strep, streptomycin.

Teow *et al*. employed the differential centrifugation technique in conjunction with antibiotics to eliminate the associated bacteria from 8 *Blastocystis* sp. isolates (Teow *et al*., 1992) (Fig. 3). The procedure involved centrifuging *Blastocystis* sp. in the logarithmic growth phase at 3000 ***g*** for 15 min, followed by resuspension in ~10 mL of phosphate-buffered saline (PBS, pH 7.0) containing 4000 *μ*g mL^−1^ ampicillin and 1000 *μ*g mL^−1^ streptomycin. Subsequently, the suspension underwent centrifugation at 1000 ***g*** for 15 min, the supernatant was discarded and 10 mL of PBS containing antibiotics was added. This entire process was repeated twice. However, limited studies have reported the axenic isolation of *Blastocystis* sp. using differential centrifugation, possibly attributed to the fragility of *Blastocystis* sp. cells (Zierdt and Williams, 1974; Teow *et al*., 1992).

#### Density gradient separation

**Figure 4.**
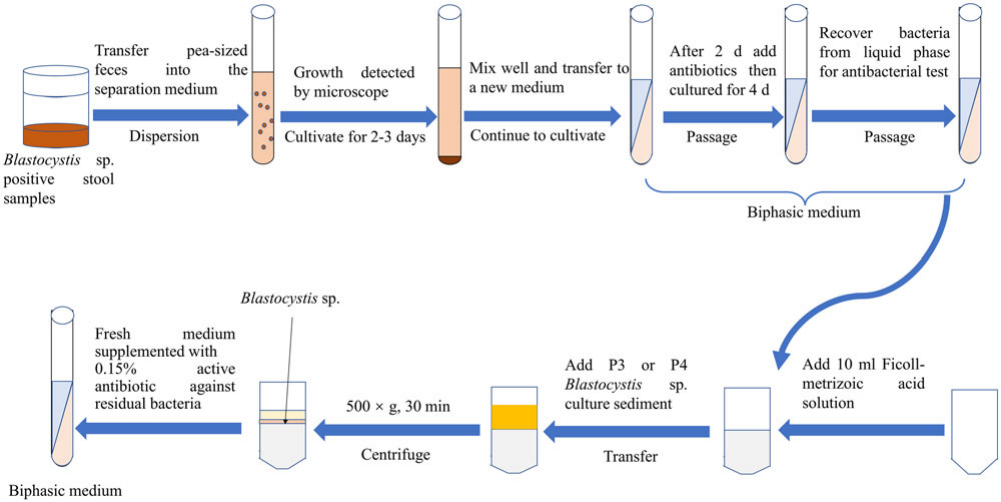
Density gradient centrifugation for the axenic isolation of *Blastocystis* sp. P, passage.

To isolate *Blastocystis* sp. from bacteria, density gradient separation has proven effective (Fig. 4). Upcroft *et al*. utilized the Ficoll–Paque gradient method to purify *Blastocystis* sp. and eliminate more than 75% of the bacteria, although complete sterility was not attained (Upcroft *et al*., 1989). Lanuza *et al*. purified *Blastocystis* sp. using the Ficoll-metrizoic acid solution and subsequently inoculated them into a fresh medium containing active antibiotics against residual bacteria, thus achieving sterility (Lanuza *et al*., 1996*b*). Defaye *et al*. reported a method for purifying *Blastocystis* sp. cysts from feces, employing the Percoll gradient method and an antibiotic mixture to reduce the number of bacteria inoculated per animal to less than 250 colonies (Defaye *et al*., 2018). See Table 2 for details.

**Table 2.** Introduction of different density gradient methods

Separation method	Separator dose (mL)	Centrifugal condition	Collection location	Effect	Reference
Ficoll–Paque separation	10	2000 ***g*** 20 min	Band at ~1 cm from the top of Ficoll–Paque	Eliminated more than 75% of the bacteria	Upcroft *et al*. (1989)
Ficoll-metrizoic acid solution separation	10	500 ***g*** 30 min	Band at a gradient of ~0.5–1 cm from the top	*Blastocystis* sp. strain was aseptic	Lanuza *et al*. (1996*b*)
Percoll separation	7	1200 ***g*** 10 min 1700 ***g*** 10 min	Between distilled water and Percoll solution	Bacterial count decreased to <250 colonies	Defaye *et al*. (2018)

#### Other axenic isolation methods

Hess *et al*. employed a micropipette, that is, micromanipulation, to isolate *Blastocystis* sp. from turkey caecal contents for cloning, offering a step towards asepsis (Hess *et al*., 2006). Shin *et al*. successfully established an axenic pure culture system for *Blastocystis* sp. using LES biphasic medium, mLES biphasic medium, liquid IMDM and solid IMDM (Shin *et al*., 2017).

Furthermore, several reports have documented the axenic culture of *Blastocystis* sp. from diverse animal species, including rats, snakes, lizards and crocodiles (Teow *et al*., 1991, 1992; Chen *et al*., 1997; Ng and Tan, 1999). Table 3 provides a summary of axenic isolation methods specific to each animal.

**Table 3.** Summary of the aseptic isolation methods from humans, rats and some reptiles

Host	Separation medium	Aseptic method	Antibiotic	Reference
Human	LES medium	Antibiotic	4000 *μ*g mL^−1^ ampicillin, 1000 *μ*g mL^−1^ streptomycin and 50 *μ*g mL^−1^ amphotericin	Zierdt and Williams (1974)
	LES medium	Antibiotic	4 g L^−1^ ampicillin, 1 g L^−1^ streptomycin and 1.5–4.0 mg L^−1^ colistin	Kukoschke and Müller (1991)
	LES medium	Antibiotic	Ampicillin, streptomycin, colistin, cefotaxime and vancomycin	Müller (1994)
	BDMM	Antibiotic combined with density gradient separation	0.4% ampicillin, 0.1% streptomycin and 0.0006% amphotericin B	Lanuza *et al*. (1996*b*)
	IMDM, 0.36% Bacto agar	Antibiotic combined with monoclonal culture	2000 *μ*g mL^−1^ claforan, 500 *μ*g mL^−1^ ampicillin, 100 *μ*g mL^−1^ streptomycin and 100 IU mL^−1^ penicillin	Ng and Tan (1999)
	Boeck–Drbohlav medium	Antibiotic combined with density gradient separation	4 mg mL^−1^ ampicillin, 1.25 mg mL^−1^ streptomycin, 0.006 mg mL^−1^ amphotericin B, 1.25 mg mL^−1^ fosfomycin, vancomycin, imipenem, ceftazidime and ciprofloxacin	Lanuza *et al*. (1996*a*)
	DMEM	Antibiotic combined with density gradient separation	1000-unit penicillin and 4 mg mL^−1^ streptomycin	Mohammad Rahimi *et al*. (2022)
	Agar medium	Antibiotic combined with monoclonal culture	100 g mL^−1^ streptomycin and 100 IU mL^−1^ penicillin	Valido and Rivera (2007)
	Jones medium	Density gradient separation	None	Chandramathi *et al*. (2010)
	MEM	Antibiotic combined with density gradient separation	Unknown	Boreham *et al*. 1992)
	Purified IMDM or 0.36% Bacto agar	Monoclonal culture (soft agar)	None	Tan *et al*. (1996*b*)
	Purified IMDM or 0.36% Bacto agar (containing sodium mercaptoacetate)	Monoclonal culture (soft agar)	None	Tan *et al*. (1996*a*)
	IMDM-HS, 2% Bacto agar	Monoclonal culture (solid agar)	None	Tan *et al*. (2000)
	LES biphasic medium, mLES biphasic medium, IMDM liquid medium, IMDM solid medium	Antibiotic combined with culture medium	Unknown	Shin *et al*. (2017)
Snake	LES medium	Antibiotic combined with differential centrifugation technology	4000 *μ*g mL^−1^ ampicillin and 1000 *μ*g mL^−1^ streptomycin	Teow *et al*. (1992)
	Boeck–Drbohlav medium	Antibiotic	4000 *μ*g mL^−1^ ampicillin and 1000 *μ*g mL^−1^ streptomycin	Teow *et al*. (1991)
Lizard	IMDM, 0.36% Bacto agar	Antibiotic combined with monoclonal culture	2000 *μ*g mL^−1^ claforan, 500 *μ*g mL^−1^ ampicillin, 100 *μ*g mL^−1^ streptomycin and 100 IU mL^−1^ penicillin	Ng and Tan (1999)
	LES medium	Antibiotic combined with differential centrifugation technology	4000 *μ*g mL^−1^ ampicillin and 1000 *μ*g mL^−1^ streptomycin	Teow *et al*. (1992)
Tortoise	LES medium	Antibiotic combined with differential centrifugation technology	4000 *μ*g mL^−1^ ampicillin and 1000 *μ*g mL^−1^ streptomycin	Teow *et al*. (1992)
Crocodile	LES medium	Antibiotic combined with differential centrifugation technology	4000 *μ*g mL^−1^ ampicillin and 1000 *μ*g mL^−1^ streptomycin	Teow *et al*. (1992)
Rat	0.3% Bacto agar with thioglycolate added	Antibiotic combined with monoclonal culture	Ampicillin, streptomycin, colistin and claforan	Chen *et al*. (1997)

## Factors affecting the axenic isolation of *Blastocystis* sp.

### Medium composition

#### Composition of the culture medium

The composition of the culture medium significantly influences the axenic isolation of *Blastocystis* sp., with components such as growth factors, glucose, mineral salt and sodium thioglycolate playing crucial roles. Zierdt and Williams (1974) axenically isolated *Blastocystis* sp. using a medium comprising 5 components: (1) boiled traditional *Blastocystis* sp. culture solution (20%); (2) traditional autoclaved *Blastocystis* sp. culture solution (20%); (3) supernatant fluid from boiled or autoclaved *Blastocystis* sp. culture solution (20%); (4) isovitalex (1%) and (5) haemin (5 *μ*g mL^−1^). Given the strict anaerobic growth requirements of *Blastocystis* sp., the addition of sodium thioglycolate to the axenic culture medium enhances anaerobic conditions, improving colony growth and yield (Tan *et al*., 1996*a*). Furthermore, the combined application of various media was conducive to the axenic culture of *Blastocystis* sp. Optimization of the media composition, such as enhancing the traditional LES biphasic medium with agar replacement protein, simplifies preparation and reduces contamination risks (Shin *et al*., 2017).

#### Culture temperature

The optimal growth temperature for *Blastocystis* sp. in axenic culture is 37°C, and attempts to purify *Blastocystis* sp. in Diamond's TPS-1 medium (The components of TPS-1 medium contained 5 g Trypticase, 10 g Panmede, 2.5 g D-glucose, 0.5 g L-cystein HCl, 0.2 g Ascorbic acid, 2.5 g NaCl, 0.3 g K_2_HPO_4_, 0.5 g KH_2_PO_4_, 50 mL Bovine serum and 435 mL Distilled water) at 28°C have been unsuccessful (Molet *et al*., 1981). Notably, *Blastocystis lapemi* sp. nov. has an optimal growth temperature of 24°C, and attempts to adapt from 24 to 37°C resulted in the gradual demise of *B. lapemi* sp. nov. above 28°C (Teow *et al*., 1991). *Blastocystis* isolated from land turtles, iguanas, crocodiles and pythons were *successfully* axenized at 24°C (Teow *et al*., 1992), and rats were axenized at 37°C (Chen *et al*., 1997). It is highly conceivable that the variation in axenic culture temperature for *Blastocystis* sp. is linked to the different body temperatures of their respective animal hosts.

#### Culture medium character

Axenic culture of *Blastocystis* sp. can be achieved using both monophasic and biphasic media (Tan *et al*., 2000). Compared with the monophasic medium, biphasic medium preparation is complicated and prone to bacterial contamination.

### Types and doses of antibiotics used

Due to *Blastocystis* sp. inhabiting the intestinal tract, the isolation and culture process often involves a mix of unknown species and drug-resistant bacteria. The combination of 1–2 antibiotics at conventional doses may prove ineffective against bacteria due to multiple drug resistance caused by antibiotic abuse (Li *et al*., 2022). In such instances, considering larger doses of drugs along with physical separation is suggested to achieve asepsis.

### Other factors affecting axenic isolation

Axenic culture may be related to the frequency of inoculation, and some bacteria will decrease with each subsequent inoculation until they disappear completely. Centrifugation is employed to separate *Blastocystis* sp. from agar, creating favourable conditions for its growth. Tan *et al*. found that emulsifying inoculum in a small amount of pre-reduced IMDM enhances the cloning process, improving colony isolation (Tan *et al*., 2000). The morphology of *Blastocystis* sp. may also influence axenic culture, with an increase in granular forms of *Blastocystis* being closely related to a significant increase in bacterial count (Zierdt and Williams, 1974).

## Applications of the axenic isolation of *Blastocystis* sp.

### Application of the axenic isolation techniques to study the pathogenicity of *Blastocystis* sp.

The application of axenic isolation techniques is crucial for studying the pathogenicity of *Blastocystis* sp., which is implicated in various gastrointestinal diseases and may play a role in irritable bowel syndrome (Ismail *et al*., 2022). Valsecchi *et al*. showed that the amoebic form of *Blastocystis* sp. causes urticaria by affecting intestinal immune homoeostasis (Valsecchi *et al*., 2004). In the study conducted by Moe *et al*., the injection of axenic *Blastocystis* sp. isolate B into mice resulted in the discovery of vacuolar and granular parasites in the caecum. Histological examination of the caecum and colon revealed significant inflammatory cell infiltration, lamina propria oedema and mucosal exfoliation (Moe *et al*., 1997). Intramuscular injection of the same strain led to extensive necrosis and interstitial oedema in the muscle, accompanied by polymorphonuclear leucocyte infiltration (Moe *et al*., 1998). Puthia *et al*. observed that the lysate of the pure cultured *Blastocystis* ST4 WRl isolate induced apoptosis of rat intestinal epithelial cell-6 (IEC-6), altered the distribution of F-actin, reduced epithelial resistance and increased epithelial permeability of the IEC-6 monolayer (Puthia *et al*., 2006). Furthermore, Ajjampur *et al*. demonstrated that axenic *Blastocystis* sp. could degrade mucin in various segments of the large intestine, consequently impacting the mucin barrier (Ajjampur *et al*., 2016). The observed pathological conditions in the host may be linked to the disruption of *in vivo* balance by specific isolates or rare subtypes (STs). Yason *et al*. reported that specific axenic isolates of *Blastocystis* ST7 could reduce the abundance of *Bifidobacterium* and *Lactobacillus* (Yason *et al*., 2019). Importantly, it is emphasized that studies on the pathogenicity of *Blastocystis* sp. ideally should be based on axenic isolation for accurate assessments.

### Application of axenic cultures in karyotype and ST analysis of *Blastocystis* sp.

To facilitate accurate karyotype and ST analysis of *Blastocystis* sp., Upcroft *et al*. recommended isolating *Blastocystis* sp. from the associated bacteria, as the presence of bacteria in non-axenic cultures could potentially mask the detection of chromosomes (Upcroft *et al*., 1989). Karyotype analysis of *Blastocystis* sp. isolated from various hosts, such as sea snakes, rats, tortoises, iguanas and pythons, revealed independent isolates, leading to the designation of distinct species, namely *B. lapemi* sp. nov., *Blastocystis ratti* sp. nov., *Blastocystis geocheloni* sp. nov., *Blastocystis cycluri* sp. nov. and *Blastocystis pythoni* sp. nov. respectively (Teow *et al*., 1991; Singh *et al*., 1996; Chen *et al*., 1997). However, Ho *et al*. found that the karyotypes of different axenic isolates of the same host were not identical, and there were marginal differences in chromosome bands (Ho *et al*., 1994).

The classification of the above-mentioned *Blastocystis* sp. species, isolated from non-human hosts, relied on electrophoretic karyotype as the criterion for defining new species. However, due to the karyotype heterogeneity of some protozoa, this standard may be insufficient to explain the species formation of *Blastocystis* sp. Host specificity and pathogenicity of different isolates have been correlated with sequence variations in small subunit-ribosomal ribonucleic acid (SSU-rRNA), leading to the division of the genus into 28 STs (Noël *et al*., 2003; Parija and Jeremiah, 2013; Martiny *et al*., 2014). Nineteen axenic isolates of *Blastocystis* sp. and 9 isolates containing bacteria were analysed using matrix-assisted laser desorption/ionization-time of flight (MALDI-TOF) mass spectrometry (Martiny *et al*., 2014). The 19 axenic isolates produced protein maps with good resolution, suggesting that MALDI-TOF mass spectrometry could effectively distinguish the STs of axenic strains (Martiny *et al*., 2014). However, the growth hindrance of *Blastocystis* sp. in the presence of bacteria and fungi resulted in correct identification for only 3 out of the 9 non-axenic isolates (Martiny *et al*., 2014).

### Application of axenic cultures for immunological studies of *Blastocystis* sp.

Current immunological studies on *Blastocystis* sp. infection primarily focus on the detection of antibodies and antigens (Sheela *et al*., 2020). The potential pathogenic mechanisms of *Blastocystis* sp. can be preliminarily elucidated through *in vitro* co-culturing of live cells, lysates, secretions and soluble antigens. Notably, since bacteria can act as antigens and potentially react with cultured cells, the success of these *in vitro* experiments relies on the use of pure cultures of *Blastocystis* sp. (Deng and Tan, 2022).

Kukoschke *et al*. reported at least 2 different peptide patterns and antigenic variants of axenic *Blastocystis* sp. (Kukoschke and Müller, 1991). Müller (1994) divided 61 axenic isolates of *Blastocystis* sp. into 4 groups using immunodiffusion, while Lanuza *et al*. (1999) divided 18 axenic strains into 3 related patterns using sodium dodecyl sulphate-polyacrylamide gel electrophoresis. The findings of the present study corroborated Kukoschke's findings. Serum obtained from mice injected with 20 *μ*g mL^−1^ soluble antigen of symptomatic and asymptomatic axenic *Blastocystis* ST3 demonstrated specific cleavage of the corresponding live *Blastocystis* sp. (Sheela *et al*., 2020). Serum obtained from mice with soluble antigen of symptomatic *Blastocystis* ST3 added to asymptomatic *Blastocystis* ST3, and vice versa, exhibited only 17% cross-reactivity, indicating significant epitope differences between symptomatic and asymptomatic *Blastocystis* ST3. These results also substantiate Kukoschke's report. However, it is essential to note that there are currently no validated cultures of axenic *Blastocystis* ST3, and Sheela *et al*. did not provide relevant evidence of the successful axenization of *Blastocystis* ST3 isolate.

*Blastocystis* sp. exerts regulatory effects on cells by modulating cytokine expression. Axenic *Blastocystis* sp. antigen induces the expression of interleukin (IL)-1*β*, IL-6 and tumour necrosis factor-alpha (TNF*-α*) in intestinal explants, colons and macrophages of mice (Lim *et al*., 2014). The axenic soluble antigen of *Blastocystis* sp. was introduced into peripheral blood mononuclear cells (PBMCs) and human colon cancer cells (HCT116) in a study by Chandramathi *et al*. (2010). The study revealed that the gene expressions of interferon-gamma (IFN*-γ*) and TNF*-α* were downregulated, while those of IL-6, IL-8 and nuclear factor kappa B (NF-κB) were upregulated in PBMCs. In HCT116, the gene expression of IFN*-γ* was significantly downregulated, while the expressions of IL-6 and NF-κB were upregulated, suggesting that the *Blastocystis* sp. antigen (at a specific concentration) has the potential to promote the growth of existing tumours or cancer cells (Chandramathi *et al*., 2010).

Recently, Deng *et al*. (2022) conducted a study where axenic *Blastocystis* ST4 was orally injected into healthy mice and dextran sodium sulphate (DSS)-induced colitis mice. The study revealed that *Blastocystis* ST4 colonization altered the composition of the intestinal bacterial community in healthy mice (without adverse effects) (Deng *et al*., 2022). Moreover, the colonization of *Blastocystis* ST4 induced T helper 2 and regulatory T cell immune responses in DSS-induced colitis mice, promoting the recovery of experimentally induced colitis mice (Deng *et al*., 2022). Under similar conditions, orally injecting axenic *Blastocystis* ST7 into DSS-induced colitis mice exacerbated the severity of colitis by increasing the proportion of pathogenic bacteria and inducing pro-inflammatory IL-17A and TNF*-α*-producing CD4+ T cells (Deng *et al*., 2023).

In a nutshell, the axenic isolation of *Blastocystis* sp. plays a crucial role in establishing a *Blastocystis* sp.–cell co-culture system *in vitro* and an animal model of *Blastocystis* sp. infection *in vivo*. These models serve as the foundation for immunological research.

### The application of axenic cultures to study the surface chemical structure and lipid biochemical analysis of *Blastocystis* sp.

Studies on the surface chemical structure of *Blastocystis* sp. aid in understanding its pathogenic potential and nutritional impact (Yason *et al*., 2018). Axenic cultures offer the advantage of avoiding bacterial interference in experiments. Keenan *et al*. conducted the pioneering analysis of lipids in 6 axenic strains of *Blastocystis* sp., revealing that the phospholipid content of all strains constituted ~39% of the total lipids (Keenan *et al*., 1992). The primary neutral lipid components were sterol esters, and the predominant polar lipid component was phosphatidylcholine across all strains. However, the study noted the challenge of determining whether the lipids were synthesized by the organisms or derived from the culture media (Keenan *et al*., 1992). Subsequent research by Keenan and Zierdt further demonstrated that axenic *Blastocystis* sp. can synthesize lipids and accumulate cholesterol and intact cholesterol esters directly from the growth medium (Keenan and Zierdt, 1994). Lanuza *et al*. employed lectin probes to identify specific carbohydrates within the surface coating of axenic *Blastocystis* sp., revealing that the structure contained *α*-d-mannose, *α*-d-glucose, *N*-acetyl-*α*-d-glucosamine, *α*-l-fucose, chitin and sialic acid (Lanuza *et al*., 1996*a*). In addition, the axenic *Blastocystis* ST7 isolate B was not directly destroyed by the antimicrobial peptide LL-37, which may be attributed to the fact that the thicker surface-coated *Blastocystis* STs are more resistant to osmotic pressure, extreme pH and oxygen exposure (Yason *et al*., 2016; Yason and Tan, 2018).

### The application of axenic cultures to study the chemotherapy of *Blastocystis* sp.

Given the individual variations in treating *Blastocystis* sp. and the absence of a specific therapeutic drug, combination drugs have been employed in clinical treatments to enhance efficacy, reduce toxicity, minimize adverse reactions and delay the onset of drug resistance. The use of sterile blastocysts can effectively evaluate the effect of drugs. Metronidazole is a commonly used drug for *Blastocystis* sp. infection, induced apoptosis-like features in axenic *Blastocystis* sp. as observed by Nasirudeen *et al*. (2004). These features included nuclear condensation, nuclear DNA fragmentation, reduced cytoplasmic volume, phosphatidylserine externalization and increased cell membrane permeability. Similarly, staurosporine, an apoptosis-inducing agent, prompted similar apoptosis-like features in *Blastocystis* sp. (Yin *et al*., 2010). *In vitro* testing of 10 antiprotozoal drugs against axenic *Blastocystis* sp. revealed the order of efficacy as emetine, metronidazole, furazolidone, trimethoprim sulphamethoxazole, 5-chloro-8-hydroxy-7-iodoquinoline (enteric violine) and pentamidine (Zierdt *et al*., 1983). However, certain *Blastocystis* sp. isolates demonstrated resistance to metronidazole, which has potential mutagenic and carcinogenic effects (Nagel *et al*., 2014; Eida *et al*., 2016). Studies on *Nigella sativa* aqueous extracts revealed their inhibitory effect on axenic *Blastocystis* sp. Eida *et al*. found that *N. sativa* aqueous extracts at concentrations of 100 and 500 *μ*g mL^−1^ exerted potent lethal effects on axenic *Blastocystis* sp. isolates *in vitro* and prevented cytopathic changes in infected mice orally inoculated with *Blastocystis* sp. *in vivo* (Eida *et al*., 2016). Yason *et al*. used the axenic *Blastocystis* isolates NUH9, WR1 and B and found good efficacy of diphenyleneiodonium chloride, auranofin and BIX 01294 trihydrochloride hydrate in treating *Blastocystis* sp. infection, particularly in the axenic *Blastocystis* ST7 isolate B, which was insensitive to metronidazole (Yason *et al*., 2018).

### Application of axenic cultures for molecular biology of *Blastocystis* sp.

The application of axenic cultures in the molecular biology of *Blastocystis* sp. has played a crucial role in addressing the controversy surrounding the association between *Blastocystis* STs and its symptomatology (Stensvold *et al*., 2007). Denoeud *et al*. took a significant step by reporting the complete genome sequence of axenic *Blastocystis* ST7 isolates from a patient in Singapore. They proposed candidate genes for the study of physiopathology, opening avenues for comparative genomics with other STs and contributing to the development of typing tools for characterizing pathogenic isolates (Denoeud *et al*., 2011).

In the post-genomic era, genetic tools have become instrumental in obtaining genetically modified cells, providing essential insights into the function of *Blastocystis* genes. Li *et al*. achieved a milestone by establishing a robust gene delivery protocol using axenic *Blastocystis* ST7 isolates. Their study involved testing ectopic protein expression with a highly sensitive nano-luciferase reporting system (Li *et al*., 2019). The study identified a promoter that includes the upstream region of the 5′ untranslated region (UTR) in legumain gene. A robust transient transfection technique was established in *Blastocystis* by combining this promoter with the legumain 3′ UTR. This breakthrough technique lays a strong foundation for further investigations into the functions of key molecules in *Blastocystis* sp.

## Conclusion

**Figure 5.**
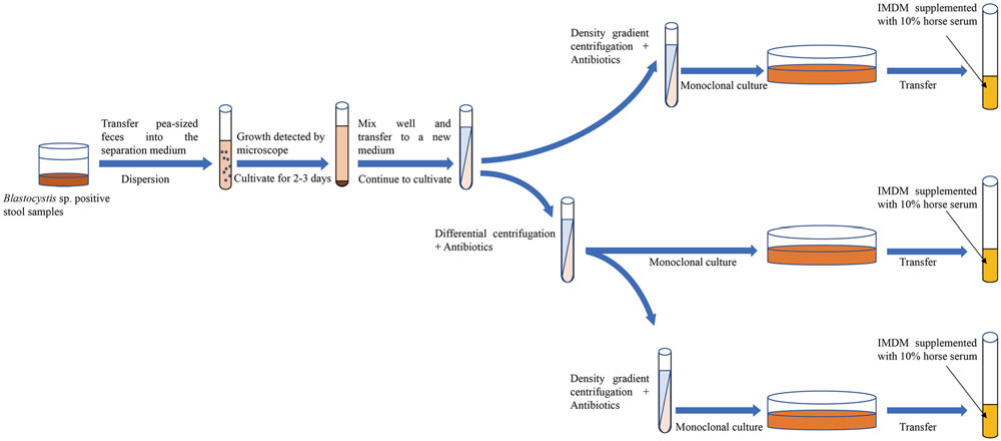
Combination of multiple methods for the axenic isolation of *Blastocystis* sp.

In summary, the axenic isolation of *Blastocystis* sp. provides a robust foundation for morphological, immunological, drug and molecular biology studies. Currently, the common approach among scholars involves the use of antibiotic treatment in conjunction with another isolation technique to achieve axenic *Blastocystis* sp. cultures. However, limited reports have explored the combined application of 3 or more axenic isolation techniques. Through an analysis of the pros and cons of various methods, it is posited that employing a combination of several axenic separation techniques proves more advantageous in obtaining pure *Blastocystis* sp. cultures (Fig. 5). Finally, it is strongly recommended that sufficient data are needed to provide supporting evidence for the successful axenization of *Blastocystis* sp. in future. This may involve presenting sterile plates to showcase the absence of bacterial growth, utilizing 16S polymerase chain reaction for bacterial detection, and supplying SSU-rRNA sequences for the confirmation of *Blastocystis* STs.

## Data Availability

The original contributions presented in the study are included in the article. Further inquiries can be directed to the corresponding author.
